# Identification of a *Pantoea* Biosynthetic Cluster That Directs the Synthesis of an Antimicrobial Natural Product

**DOI:** 10.1371/journal.pone.0096208

**Published:** 2014-05-05

**Authors:** Alyssa M. Walterson, Derek D. N. Smith, John Stavrinides

**Affiliations:** Department of Biology, University of Regina, Regina, Saskatchewan, Canada; Agriculture and Agri-Food Canada, Canada

## Abstract

Fire Blight is a destructive disease of apple and pear caused by the enteric bacterial pathogen, *Erwinia amylovora*. *E. amylovora* initiates infection by colonizing the stigmata of apple and pear trees, and entering the plants through natural openings. Epiphytic populations of the related enteric bacterium, *Pantoea*, reduce the incidence of disease through competition and antibiotic production. In this study, we identify an antibiotic from *Pantoea ananatis* BRT175, which is effective against *E. amylovora* and select species of *Pantoea*. We used transposon mutagenesis to create a mutant library, screened approximately 5,000 mutants for loss of antibiotic production, and recovered 29 mutants. Sequencing of the transposon insertion sites of these mutants revealed multiple independent disruptions of an 8.2 kb cluster consisting of seven genes, which appear to be coregulated. An analysis of the distribution of this cluster revealed that it was not present in any other of our 115 *Pantoea* isolates, or in any of the fully sequenced *Pantoea* genomes, and is most closely related to antibiotic biosynthetic clusters found in three different species of *Pseudomonas*. This identification of this biosynthetic cluster highlights the diversity of natural products produced by *Pantoea*.

## Introduction

Fire blight is a destructive disease known to plague many rosaceous plants, in particular apple and pear plants [1], [2], [3]. It has been a threat to the cultivation of these crop plants in various parts of the world, including North America and Europe [4], with first reports appearing as early as 1790 in North America [5]. The causative agent, *Erwinia amylovora*, is an epiphytic bacterium that relies on nutrients from the plants for survival [6]. The site of colonization is generally the stigmata of immature apple and pear blossoms, which provide a moist environment, rich in nutrients [4]. *E. amylovora* enters the plants through natural openings and spreads to various parts of the plants through the vascular system, allowing for widespread colonization [7]. Once *E. amylovora* is established, disease symptoms such as flower necrosis and fruit rot develop, ultimately resulting in major losses [7].

Methods of control for *E. amylovora* have been the subject of many studies. Initially, antibiotics like streptomycin were shown to be an effective means of controlling bacterial colonization when applied to plants during early bloom [8]. Long term use led to streptomycin resistant populations of *E. amylovora* [8], [9], which was a cause for concern in medical communities using the antibiotic for therapeutic purposes [8], [9]. Recent studies have shown that the application of the bacterial antagonist, *Pantoea agglomerans* (formerly *Erwinia herbicola*), to apple and pear trees in early bloom may help to reduce the disease caused by *E. amylovora* [1], [10], [11], [12]. *Pantoea vagans* C9-1 and *P. agglomerans* E325 have been commercialized as the active agents in BlightBan C9-1 and Bloomtime Biological, respectively [13], and are currently being used in several parts of the world as biocontrol agents for fire blight [14]. The antagonists not only compete for sites of colonization and plant nutrients, but also produce antibiotics that prevent the colonization of *E. amylovora* [13].

*Pantoea* isolates have been shown to produce a variety of antibiotics, such as pantocins [15], [16], [17], [18], herbicolins [19], [20], microcins [21], [22], [23], and phenazines [24], several of which target amino acid biosynthesis genes in *E. amylovora*. These antibiotics have been classified into five distinct groups based on whether they are effective in the presence of specific amino acids [25]. Group I antibiotics, which include pantocin A, herbicolin O, and microcin MccEh252, lose their toxicity in the presence of L-histidine, while groups II (pantocin B), III, and IV are neutralized in the presence of L-arginine, L-lysine, and L-asparagine, respectively. Interestingly, Group V (herbicolin I) is unaffected by the presence of any amino acid [25]. The specific targets of several of these have been determined. Pantocin B directly targets N-acetylornithine transaminase, the last step in arginine biosynthesis [26], while pantocin A acts against *E. amylovora* by inhibiting L-histidinol phosphate aminotransferase, a critical step in histidine biosynthesis, resulting in a histidine deficiency [2], [27].

In this study, we identify an antibiotic from *Pantoea ananatis* strain BRT175 (*Pan*BRT175) that is effective against *E. amylovora*, which we have called *Pantoea* Natural Product 1 (PNP-1). We evaluate the impact of nutritional changes on production, including whether PNP-1 activity is affected by amino acids. We demonstrate that the activity appears to be relatively narrow spectrum affecting *Erwinia* and *Pantoea*, and that resistance in *Pantoea* appears to have evolved in the lineage leading to the *Pantoea agglomerans* and *Pantoea eucalyptii* groups. We also identify the biosynthetic cluster responsible for PNP-1 production, and evaluate its distribution in *Pantoea* and other species.

## Materials and Methods

### Bacterial Strains

All strains used were cultured and maintained on lysogeny broth (LB) media (Table 1). *Pantoea* and *Erwinia* strains were incubated at 30°C. *E. coli* strains VPE42 (pBSL118) and HB101 (RK600), *Staphylococcus aureus* K1-7, *Pseudomonas aeruginosa* ATCC 27853, *Streptococcus mutans* UAIS9:wt, and *Lactococcus lactis* were incubated at 37°C. Where appropriate, antibiotics were supplemented in the following final concentrations: kanamycin, 50 µg/ml, chloramphenicol, 50 µg/ml, and rifampicin, 50 µg/ml.

**Table 1:** List of isolates used in this study.

Species	Isolation Source/Genotype	Source
***Enterobacter sp.***		
TX1	human, CF sputum	[59]
***Erwinia amylovora***		
EA321	hawthorn	[59]
***Escherichia coli***		
DH5a	*fhuA2 lac(del)U169 phoA glnV44 Φ80′ lacZ(del)M15 gyrA96 recA1 relA1 endA1 thi-1 hsdR17*	Dr. David Guttman, University of Toronto
HB101 (RK600)	conjugative plasmid	Dr. David Guttman, University of Toronto
VPE42 (pBSL118)	mini-Tn5 transposon	[60]
***Lactococcus lactis***		
HD1		Heather Dietz, University of Regina
***Pantoea agglomerans***		
83	wheat	ICMP
788	green bean	ICMP
1512	green bean	ICMP
1574	unidentified	ICMP
3581	oat seed	ICMP
5565	soybean	ICMP
7373	onion	ICMP
7612	grass grub	ICMP
12531	Gypsophila (Baby's Breath)	ICMP
12534	human, knee laceration	ICMP
13301	Golden Delicious apple	ICMP
17124	olive	ICMP
770398	human, female, blood	[59]
240R	pear flower	[59]
308R	pear flower	[59]
B015092	human, female, urine midstream	[59]
B016395	human, female, superficial wound	[59]
B025670	human, female, superficial wound	[59]
B026440	human, male, superficial wound	[59]
BB834250	human, female, sputum, aortic aneurysm	[59]
DB522094	human, elbow sore	[59]
DC432	maize	[61]
DC434	maize	[61]
DC556	Gypsophila (Baby's Breath)	[61]
Eh318	apple leaf	CUCPB 2140; Dr. Brion Duffy [18]
G4032547	human, ear	[59]
H42501	human, male, blood	[59]
SN01080	slug	[59]
SN01121	bee	[59]
SN01122	bee	[59]
SN01170	caterpillar	[59]
SP00101	raspberry	[59]
SP00202	apple	[59]
SP00303	raspberry	[59]
SP01201	strawberry leaf	[59]
SP01202	strawberry leaf and stem	[59]
SP01220	healthy rose bush	[59]
SP01230	Virginia creeper leaves and stem	[59]
SP02022	thistle	[59]
SP02230	diseased tree leaf	[59]
SP02243	unidentified tree	[59]
SP03310	diseased tree leaf	[59]
SP03383	diseased maize leaf	[59]
SP03412	diseased bean leaf	[59]
SP04010	tomato leaf	[59]
SP04011	tomato leaf	[59]
SP04021	tomato leaf	[59]
SP04022	tomato leaf	[59]
SP05051	tomato leaf	[59]
SP05052	tomato leaf	[59]
SP05061	tomato leaf	[59]
SP05091	tomato leaf	[59]
SP05092	tomato leaf	[59]
SP05120	diseased maize leaf	[59]
SP05130	diseased maize stamen	[59]
SS02010	soil - ground squirrel burrow	[59]
SS03231	soil - ground squirrel burrow	[59]
TX10	human, CF sputum	[59] [62]
***Pantoea ananatis***		
15320	rice	[59]
17671	rice	[59]
26SR6	maize leaf	Dr. Steven Lindow, UC Berkeley [59]
B7	maize, rifR derivative of M232A	Dr. Steven Lindow, UC Berkeley [59]
BRT175	strawberry	Dr. Gwyn Beattie, Iowa State [59] [28]
BRT98	strawberry	Dr. Steven Lindow, UC Berkeley [59]
Cit30-11	naval orange leaf	Dr. Steven Lindow, UC Berkeley [59]
M232A	maize	Dr. Steven Lindow, UC Berkeley [59]
***Pantoea anthophila***		
1373	balsam	[59]
***Pantoea brenneri***		
91151	human	[59]
B011483	human, female, superficial wound	[59]
B014130	human, male, superficial wound	[59]
B016381	human, female, groin	[59]
B024858	human, female, breast abscess	[59]
***Pantoea calida***		
B021323	human, female, urine midstream	[59]
BB957621A1	human, male, CAPD dialysate, peritonitis	[59]
BB957621A2	human, male, CAPD dialysate, peritonitis	[59]
BB957621B1	human, male, CAPD dialysate, peritonitis	[59]
BB957621B2	human, male, CAPD dialysate, peritonitis	[59]
BB957621C1	human, male, CAPD dialysate, peritonitis	[59]
BB957621C2	human, male, CAPD dialysate, peritonitis	[59]
***Pantoea conspicua***		
B011017	human, female, superficial wound	[59]
***Pantoea dispersa***		
625	sorghum	ICMP
M1657A	human, male, blood	[59]
M1657B	human, male, blood	[59]
***Pantoea eucalyptii***		
299R	pear flower	Dr. Steven Lindow, UC Berkeley
B011489	human, female, superficial wound	[59]
F9026	human, male, blood	[59]
SM03214	goose feces	[59]
SP02021	thistle leaf	[59]
SP03372	diseased maize leaf	[59]
SP03391	diseased bean leaf	[59]
SP04013	tomato leaf	[59]
***Pantoea eucrina***		
6686	human, headache	[59]
TX5	human, blood	[59]
TX6	human, blood	[59]
***Pantoea septica***		
81828	human, post hemicholectomy	[59]
101150	human	[59]
062465A	human, cerebellar CVA (stroke)	[59]
062465B	human, cerebellar CVA (stroke)	[59]
091957A	human, renal failure	[59]
091957B	human, renal failure	[59]
B016375	human, female, finger	[59]
BB350028A	human, female, blood culture, fever	[59]
BB350028B	human, female, blood culture, fever	[59]
BE528629	human, peritoneal dialysis	[59]
G2291404	human	[59]
G3271436	human, urine	[59]
G4071105	human, urine	[59]
M1517	human, female, blood	[59]
M41864	human, female, blood	[59]
TX3	human, blood	[59]
TX4	human, blood	[59]
VB38951A	human, female, blood culture, sore throat	[59]
VB38951B	human, female, blood culture, sore throat	[59]
X44686	human, female, blood	[59]
***Pantoea stewartii***		
626	maize	ICMP
DC283	maize	[63]
***Pseudomonas aeruginosa***		
ATCC 27853	clinical	Heather Dietz, University of Regina
***Pseudomonas syringae*** ** pv. s** ***yringae***		
ES4326	radish pathogen	Dr. David Guttman, University of Toronto
***Staphylococcus aureus***		
K1-7	clinical	Dr. Chris Yost, University of Regina
***Streptococcus mutans***		
UAIS9:wt	clinical	Heather Dietz, University of Regina

### Effects of Nutrition on Antibiotic Activity

The antibiotic activity of *Pan*BRT175 was evaluated using the agar overlay method on *E. coli* minimal media with peptone concentrations of 2.5 mg/ml, 5.0 mg/ml, 10.0 mg/ml, and 15.0 mg/ml, and tryptone concentrations of 5.0 mg/ml, 10.0 mg/ml, 15.0 mg/ml, and 20.0 mg/ml, both with and without glucose. Antibiotic activity was also evaluated in the presence of the following amino acids: alanine, β-alanine, asparagine, aspartic acid, arginine, cystine, glutamic acid, glutamine, glycine, histidine, isoleucine, leucine, lysine, methionine, orinithine, phenylalanine, proline, serine, threonine, tryptophan, tyrosine, and valine. All amino acids were obtained from Sigma-Aldrich®, except for β-alanine, asparagine, and arginine, which were obtained from Alfa Aesar®. The amino acids were added into separate 5X Glycerol Salt Solutions at a concentration of 1.5 mg/ml, which was then added into the top layer.

### Transposon Mutagenesis and Screening

Transposon mutagenesis of *Pan*BRT175 was carried out with a triparental mating involving the helper *E. coli* HB101 (RK600) and the mini-Tn5 donor *E. coli* VPE42 (pBSL118). Overnight LB broth cultures of each strain were aliquoted in 1 mL volumes, centrifuged at 13,500 g for 1 minute, and resuspended in 100 µL of 10 mM Mg_2_SO_4_. All three were combined, vortexed briefly, and three 100 µL volumes were spotted on to a single LB agar plate, which was incubated overnight at 30°C. Following 16-24 hours of incubation, small samples of each triparental spot were spread on to individual LB agar plates containing kanamycin and incubated at 30°C for 48 hours.

Screening for *Pan*BRT175 mutants was performed using an agar overlay method. The bottom agar was *E. coli* Minimal Medium (per liter: 0.25 g yeast extract, 1.72 g KH_2_PO_4_, 4.0 g K_2_HPO_4_, 0.5 g NaCl, 0.2 g sodium citrate, 2.0 g (NH_4_)_2_SO_4_, 0.002 g MgSO_4_*7H_2_O, 20 mL glycerol) with a top layer of 0.9% agar containing 5X Glycerol-Arginine Salt Solution (per 100 mL: 5.57 g K_2_HPO_4_, 2.25 g KH_2_PO_4_, 0.06 g MgSO_4_*7H_2_O, 10.0 g glucose, 0.025 g nicotinic acid, 0.15 g L-asparagine) seeded with *E. amylovora* EA321, resuspended in 300 µL of 5 mM K_2_HPO_4_. *Pan*BRT175 mutants were picked from the triparental spread plates based on pigmentation and spotted on to the *E. coli* minimal media plate. The plates were incubated at 30°C overnight. Mutants that did not inhibit the EA321 indicator in the agar overlay were selected, retested, and cultured on LB agar containing kanamycin.

### Inverse PCR, Sequencing, and Sequence Analysis

All selected *Pan*BRT175 mutants were inoculated into 3 mL of LB broth and incubated overnight at 30°C. Genomic DNA was then extracted using E.Z.N.A. Bacterial DNA Kit (Omega Bio-Tek®). The genomic DNA was digested in a reaction containing 5 units HincII (New England Biolabs), 0.2 µL 10x Buffer 3, 2.0 µL BSA (10 mg/ml), 13.8 µL dH_2_O, 2.0 µL gDNA (280-440 ng/µL) and a unimolecular ligation performed in a reaction containing 3 units T4 DNA Ligase (NEB), 162.0 µL dH_2_O, 20.0 µL T4 DNA Ligase Buffer, and 15.0 µL digested gDNA. Samples at both steps were cleaned using E.Z.N.A. Purification Kit (Omega Bio-Tek®). An inverse PCR was then performed (8.6 µL dH_2_O, 0.2 µL EconoTaq DNA Polymerase (Lucigen) (5 U/µL), 0.5 µL forward primer (50 µM), 0.5 µL reverse primer (50 µM), 2.0 µL 10X EconoTaq Buffer, 1.2 µL 25 mM MgCl_2_, 2.0 µL dNTPs (200 µM each), 5.0 µL template DNA) and resulting samples were purified directly, or extracted from the gel using Omega E.Z.N.A. Gel Extraction Kit. Sequencing was performed by Eurofins MGW Operon (Huntsville, AL, USA).

### Genetic and Genomic Analyses

Mutant sequences were queried against GenBank at NCBI using the Basic Local Alignment Search Tool (BLAST). Mutant sequences were also queried against a draft sequence of the *Pan*BRT175 genome (Accession: ASJH00000000) [28] using standalone BLAST to identify the location of the gene cluster. Once identified, each gene was queried against the draft genomes of *Pantoea septica* X44686, *Pantoea agglomerans* Eh318, *Pantoea ananatis* BRT98, *Pantoea agglomerans* TX10, *Pantoea eucalyptii* F9026, *Pantoea ananatis* 15320, *Pantoea agglomerans* DC432, *Pantoea dispersa* 625, *Pantoea eucalyptii* 299R, *Pantoea eucalyptii* SP03391, *Pantoea agglomerans* SP04022, *Pantoea eucalyptii* SP03372, *Pantoea eucalyptii* SP04013, *Pantoea eucalyptii* SP02021, *Pantoea agglomerans* SP00101, *Pantoea agglomerans* VB39851-A, *Pantoea calida* B021323, *Pantoea eucalyptii* B011489, *Pantoea agglomerans* B025670, *Pantoea brenneri* B024858, *Pantoea brenneri* B016381, *Pantoea dispersa* M1657A, and *Pantoea calida* BB957621-B2 using standalone BLAST with an e-value cutoff of 0.0001 to identify even the most divergent potential homologs. The NCBI Conserved Domain Database (CDD) was used to assign each protein to a family.

### *Pantoea agglomerans* Eh318 Genome Sequencing

Total DNA was sequenced using Illumina HiSeq 2000, 100-bp paired-end sequencing, resulting in 16,055,112 reads, with an average Phred quality score of 32. ABySS version 1.3.5 was used for de novo paired-end assembly using the default parameters and an optimized k-mer value of 87. This resulted in 51 contigs with an N_50_ of 376,883 bp and an estimated genome size of 5,036,004 bp at 319X coverage. Contigs that were 200 bp or larger (37 total) were submitted to the NCBI Prokaryotic Genome Automatic Annotation Pipeline version 2.0. The genome has been deposited under accession number AXOF00000000.

### Distribution of the PNP-1 Cluster in *Pantoea*

PCR primers (Table 2) specific to *pnp1A*, *pnp1C*, *pnp1D*, and *pnp1F* were used to examine the distribution of the biosynthetic cluster in *Pantoea*. Colony PCR was performed for each *Pantoea* isolate by toothpick inoculation of a single colony into a PCR reaction containing 13.6 µL dH_2_O, 0.2 µL EconoTaq DNA Polymerase (5 U/µL), 0.5 µL forward primer (50 µM), 0.5 µL reverse primer (50 µM), 2.0 µL 10X EconoTaq Buffer, 1.2 µL 25 mM MgCl_2_, and 2.0 µL dNTPs (200 µM each), on each *Pantoea* strain (Table 1).

**Table 2:** List of primers used in this study.

Gene	Primer	Sequence	Length	Annealing Temp
*pnp1A*	GntR+11	GTGCTGTCGATACAGACGGCGCAT	24	68.0
	GntR-700	GACGTGATCCTGCGGGCTTACTGTC	25	69.5
*pnp1C*	07851+59	GCAAGCTCAACCGTAGGATATTCTC	25	64.6
	07851-680	AATTAGACTGTCAAGAGAGAATGGT	29	66.0
*pnp1D*	Carb+64	ACAAGCACTGAGCAGCCCGTCAGC	25	69.5
	Carb-836	TGCAGCATCAGTGACTGGTGAGAGA	25	66.2
*pnp1F*	07881+95	GATCCGGGTGATGCGTGGCCAGAG	25	72.8
	07881-555	TGAAACAGCGGTGATCCGGTTCGT	25	66.2
mini-Tn5 transposon	npt-41	AGCCGAATAGCCTCTCCACCCAAG	24	68.0
	npt+772	TTCGCAGCGCATCGCCTTCTATC	23	66.3

## Results and Discussion

### Effects of Nutrition on Antibiotic Activity

Antibiotic production assays of *Pan*BRT175 were carried out on *E. coli* minimal medium supplemented with glucose, which resulted in a zone of inhibition (ZOI) in a lawn of *E. amylovora*. To determine how antibiotic production and/or activity was affected by nutritional changes, the assay was attempted on the rich medium, LB. The ZOI was reduced to approximately half the size of that formed on minimal medium with glucose (Figure S1). The assay was repeated with varying concentrations of either peptone or tryptone in minimal salt medium, with and without glucose. Antibiotic production was greatest at the lowest concentrations of 2.5 mg/ml peptone and 5.0 mg/ml tryptone (approximately double the ZOI formed on minimal medium) whether or not glucose was present. At the higher concentrations, 10.0 mg/ml peptone, 15.0 mg/ml peptone and 15.0 mg/ml tryptone, and in the presence of glucose, antibiotic production was still approximately double, but the edge of the ZOI was less defined (Figure S1). The remaining peptone and tryptone concentrations with and without glucose had similar PNP-1 production as minimal medium with glucose. In general, these results suggest that PNP-1 biosynthesis is not repressed significantly under nutrient-rich conditions, as has been reported for several antibiotic biosynthetic pathways [29]. Antibiotics like streptothricin from *Streptomyces lavendulae* and streptomycin from *Streptomyces griseus*, are also produced in abundance on tryptone-starch and nutrient medium, respectively [30]. Micronutrients and salts can also alter antibiotic production with phenazine production and accumulation in *Pseudomonas* being affected by the presence of boric acid, iron and magnesium sulfate [31].

*Pantoea* antibiotics target specific amino acid biosynthesis pathways, and have been traditionally grouped by their loss of effectiveness in the presence of certain amino acids. To determine whether the activity of this antibiotic could be neutralized in the presence of amino acids, each amino acid was supplemented into the agar overlay on minimal salt medium. Antibiotic activity against *E. amylovora* was not affected by any of the tested amino acids, which is consistent with the Group V antibiotics, such as herbicolin I [19], [20]. Interestingly, some *E. amylovora* colonies were able to spontaneously acquire resistance to PNP-1, suggesting that a mutation in a single gene can result in antibiotic resistance. Retests using an agar overlay assay confirmed that the isolated spontaneous mutants were indeed resistant, as no ZOI formed.

### Spectrum of Antibiotic Activity

The antibiotic produced by *Pan*BRT175 showed a narrow spectrum of activity inhibiting *E. amylovora*, but not *S. aureus, P. aeruginosa, S. mutans*, *E. coli*, or *L. lactis* (Table 3). We extended this testing to 30 isolates of *Pantoea* representing 11 different species. Of these, 20 isolates were susceptible, while all resistant *Pantoea* strains fell into the closely related *P. agglomerans* and *P. eucalyptii* groups (Table 3). This suggests that resistance to PNP-1 was acquired by a common ancestor of these sister species [32], and that resistance is not necessarily directly tied to the biosynthetic cluster. The antibiotic showed activity against both clinical and environmental *Pantoea* isolates in the other species groups. Both *P. ananatis* and *P. stewartii* are known plant pathogens [33], [34], [35], [36], [37], [38], [39], [40], [41], [42], [43], [44], and so PNP-1 has the potential to be used in its purified form for control of these strains.

**Table 3:** Evaluation of resistance to PNP-1 across non-*Pantoea* and *Pantoea* species.

Strain	Source	Resistant (R)/Susceptible (S)
***Erwinia amylovora***		
EA321	Environmental	S
***Escherichia coli***		
DH5a	Lab strain	R
***Lactococcus lactis***		
HD1	Environmental	R
***Pantoea agglomerans***		
1512	Environmental	R
83	Environmental	R
5565	Environmental	R
Eh318	Environmental	R
DC432	Environmental	R
B016395	Clinical	R
B025670	Clinical	R
B026440	Clinical	R
TX10	Clinical	R
SP05130	Environmental	R
SS02010	Environmental	R
SP05120	Environmental	R
SP04022	Environmental	R
***Pantoea eucalyptii***		
299R	Environmental	R
F9026	Clinical	R
B011489	Clinical	R
SP03372	Environmental	R
SP02021	Environmental	R
SP04013	Environmental	R
SP03391	Environmental	R
***Pantoea conspicua***		
B011017	Clinical	S
***Pantoea brenneri***		
B016381	Clinical	S
B024858	Clinical	S
***Pantoea anthophila***		
1373	Environmental	S
***Pantoea stewartii***		
DC283	Environmental	S
262	Environmental	S
***Pantoea ananatis***		
BRT98	Environmental	S
15320	Environmental	S
17671	Environmental	S
Cit30-11	Environmental	S
M232A	Environmental	S
B7	Environmental	S
26SR6	Environmental	S
***Pantoea calida***		
B021323	Clinical	S
BB957621-B2	Clinical	S
***Pantoea septica***		
B016375	Clinical	S
VB38951-A	Clinical	S
X44686	Clinical	S
***Pantoea dispersa***		
M1657A	Clinical	S
625	Environmental	S
***Pantoea eucrina***		
6868	Clinical	S
***Pseudomonas aeruginosa***		
ATCC 27853	Clinical	R
***Staphylococcus aureus***		
K1-7	Clinical	R
***Streptococcus mutans***		
UAIS9:wt	Clinical	R

### Identification of the Biosynthetic Cluster

Transposon mutagenesis was used to identify the antibiotic biosynthetic cluster in *Pan*BRT175. Approximately 5000 mutants were screened using the agar overlay method, resulting in 41 independent mutants confirmed to be impaired in antibiotic production and/or secretion. We analyzed 29 of these mutants, and in four of them, the transposon had inserted into the disrupted carbamoyl-phosphate synthase large chain (*carB*) and phosphoribosylformylglycinamidine synthase (*purL*) genes, which are necessary components in purine and pyrimidine metabolism [45], [46] (Table 4). In the other 25 mutants, the transposon had inserted independently in six genes that were part of a 8.2 kb seven-gene cluster in the *Pan*BRT175 genome [28] (Table 4).

**Table 4:** Recovered *P. ananatis* BRT175 mutants defective in PNP-1 production/export.

Mutant	Gene	Organism	Putative protein	Conserved domains	Length (nt)	% Similarity	Accession
5, 6, 18	*pnp1A*	*P. ananatis* BRT175	GntR Transcriptional Regulator	Aspartate Aminotransferase Family, cd00609; Winged helix-turn-helix, cd07377.	1404		ERM15323
		*P. syringae pv. maculicola* ES4326			1317	48	EGH58727
							
9, 16, 26	*pnp1B*	*P. ananatis* BRT175	Hypothetical protein	SGNH/GDSL hydrolase Family, pfam13472	972		ERM14577
		*P. syringae pv. maculicola* ES4326			993	75	EGH58728
							
4	*pnp1C*	*P. ananatis* BRT175	Hypothetical protein	N/A	2111		ERM15479
		*P. syringae pv. maculicola* ES4326			2202	61	EGH58729
							
2, 3, 8, 10, 14, 22, 24, 27, 28	*pnp1D*	*P. ananatis* BRT175	Carbamoyltransferase	NodU/CmcH Family, cl12209	1830		ERM15480
		*P. syringae pv. maculicola* ES4326			927	70	EGH58732
							
1, 7, 11, 12, 15, 17, 25	*pnp1E*	*P. ananatis* BRT175	Hypothetical Protein	Aspartate Aminotransferase (AAT) Family, cl00321	1474		ERM15482
		*P. syringae pv. maculicola* ES4326			1317	72	EGH58734
							
13,23	*pnp1F*	*P. ananatis* BRT175	Hypothetical Protein	Formyltransferase Core Superfamily, cd08369	683		ERM15483
		*P. syringae pv. maculicola* ES4326			600	70	EGH58735
							
N/A	*pnp1G*	*P. ananatis* BRT175	MFS Transporter	MFS Superfamily, cd06174	560		ERM15324
		*P. syringae pv. maculicola* ES4326			627	61	EGH58736
							
							
19	*carB*	*P. ananatis* BRT175	Carb3	ATP-grasp Superfamily, cl17255	3230		ERM15221
		*P. ananatis* LMG 5342			3228	83	YP_005197068
							
20, 21, 29	*purL*	*P. ananatis* BRT175	PurL	PurM-like Superfamily, cl10019	3890		ERM12406
		*P. ananatis* LMG 5342			3888	97	AER31669

The first gene identified is the divergently transcribed *pnp1A*, which belongs to COG1167 (ARO8), a family whose members contain both a winged helix-turn-helix domain of the GntR family of transcriptional regulators (Conserved Domain Database Accession: cl17414), as well as an aspartate aminotransferase domain (CDD Accession:cl00321). The GntR transcriptional regulators have been implicated in bacterial growth and development, as well as antibiotic production [47]. Interestingly, antibiotic biosynthesis in one of the recovered *pnp1A* mutants could be restored with the exogenous supplementation of histidine. Transposon insertion in this mutant occurred in the aminotransferase region, possibly indicating that histidine is a PNP-1 precursor, and that the product of this gene plays a role in regulating histidine availability. The supplementation of histidine would have bypassed the requirement for this locus, thereby restoring antibiotic biosynthesis. The *pdxR* regulator of *Streptomyces venezuelae*, which also contains both an aminotransferase and helix-turn-helix domain was shown to complement pyridoxal (vitamin B_6_) auxotrophy by regulating the availability of pyridoxal phosphate and related co-enzymes [48]. None of the other 29 PNP-1 mutants could be rescued by histidine, or any other amino acids.

**Figure 1:**
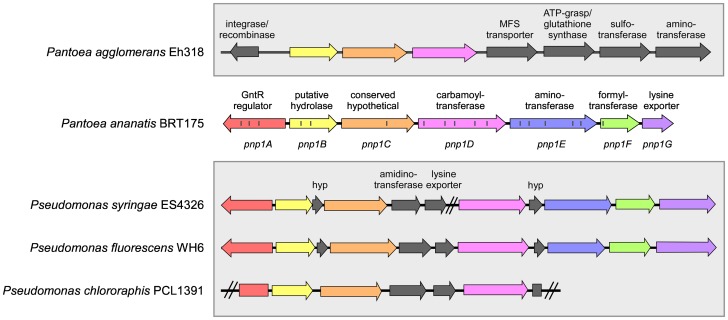
The composition and organization of the 8.2-1 antibiotic biosynthetic cluster in *P. ananatis* BRT175 (Accession: ASJH00000000) . The insertion sites of the mini-Tn5 transposon for mutants generated by transposon mutagenesis are indicated with lines within the cluster. Homologous biosynthetic gene clusters of *P. syringae pv. maculicola* ES4326 (11.6 kb) (Accessions: AEAK01000141, AEAK01000142), *P. fluorescens* WH6 (11.6 kb) (Accession: AEAZ01000041), and *P. chlororaphis* PCL1391 (7.5 kb) (Accession: DQ367408) are shown. A gene cluster in *P. agglomerans* Eh318 (10kb) (Accession: AXOF00000000) having several homologous genes is shown. Gene colours indicate homology between the biosynthetic clusters. Cluster in *P. syringae pv. maculicola* ES4326 overlaps two contigs.

*pnp1B, pnp1C*, and *pnp1D* are the next three genes of the predicted biosynthetic cluster (Figure 1). *pnp1B* belongs to the SGNH/GDSL family of hydrolases (CDD Accession: cl01053), which include esterases and lipases that have known activity towards esters, acyl-CoAs, and amino acid derivatives. Applications for these hydrolytic enzymes include synthesis of pharmaceuticals, including phospholipase A and B [49]. *pnp1C* is a 2111 bp-long predicted coding region; however, it has no identifiable protein domain that could indicate biochemical function. Finally, *pnp1D* codes for a carbamoyltransferase of the NodU/CmcH family (CDD Accession: cl12209, pfam02543), which is responsible for post-transcriptional modifications of late gene products [50]. Carbamoyltransferases are key enzymes in β-lactam antibiotic production in certain species of *Rhizobium, Bradyrhizobium*, and *Streptomyces* [50]. Homologs of these three genes were identified in the gene clusters of *Pseudomonas syringae* pv. maculicola ES4326, *Pseudomonas fluorescens* WH6, and *Pseudomonas chlororaphis* PCL1391, as well as *P. agglomerans* Eh318 (discussed later) (Figure 1, Table 4).

The product of *pnp1E* falls under the aspartate aminotransferase (AAT) superfamily (fold type I) of pyridoxal phosphate (PLP)-dependent enzymes (Accession: cl00321), members of which are known to play a role in transamination, racemization, decarboxylation, and various other side chain reactions [51]. The product of *pnp1F* belongs to the formyltransferase, catalytic core domain superfamily (CDD Accession: cl00395), which is known to be involved in purine biosynthesis, formate biosynthesis, translation initiation, and modification of lipid A, leading to resistance to cationic antimicrobial peptides and clinical antimicrobials [51].

The final annotated gene in the *Pan*BRT175 cluster, *pnp1G*, is a member of the MFS transporter family that includes the LysE type translocator family (pfam01810) and the RhtB homoserine/threonine transport family (TIGR00949, COG1280). LysE exporters have been noted to transport L-lysine, cadmium, and various quaternary amines in bacterial species such as *E. coli*, *Mycobacterium tuberculosis*, and *Bacillus subtilis* [52], while the RhtB protein family has been proposed to be involved in the excretion of metabolites [53]. This is consistent with the fact that transposon insertion mutants were not recovered for this gene, since disruption could result in metabolite accumulation and lethality. Alternatively, this could have been a consequence of our non-saturating genetic screen.

### Distribution of the PNP-1 Biosynthetic Cluster

To determine whether this antibiotic cluster was present in other *Pantoea* strains, a PCR-based survey was conducted on four of the seven genes (*pnp1A, pnp1C, pnp1D*, and *pnp1F*). A total of 117 strains were evaluated, all of which were negative for all four genes in the antibiotic cluster. Standalone BLASTs were also performed for each gene in the PNP-1 cluster against a draft genome sequence of *P. agglomeran*s Eh318, an isolate known to produce at least two antibiotics, pantocins A and B, which are effective against *E. amylovora* [18]. Interestingly, homologs of *pnp1B, pnp1C*, and *pnp1D* were identified as the first genes in a seven-gene 10 kb cluster in Eh318, while the remaining genes of the cluster include an MFS transporter, ATP-grasp/glutathione synthase, sulfotransferase, and aminotransferase (Figure 1). Although this suggests that this Eh318 gene cluster may direct the synthesis of another natural product, there is no evidence that this product is antimicrobial.

Because the PNP-1 cluster was unique and not found complete in any other isolate in our collection, we examined its distribution in other species. We queried GenBank and identified similar gene clusters in *Pseudomonas syringae pv*. maculicola ES4236, *Pseudomonas fluorescens* WH6 and *Pseudomonas chlororaphis* PCL1391 (Table 4). The clusters in ES4326 and WH6 were 11.6 kb in size and consisted of a total of eleven genes, seven of which corresponded to those found in the PNP-1 biosynthetic cluster (Figure 1). Four genes present in the *Pseudomonas* clusters that are annotated as hypothetical protein, inosamine-phosphate amidinotransferase, lysine exporter, and hypothetical protein, are absent from the PNP-1 cluster (Figure 1). Since there was substantial similarity between PNP-1 and clusters of PC1391, ES4326 and WH6, we performed some preliminary experiments on ES4326 to evaluate whether it can also antagonize *Erwinia*. An overlay assay with ES4326 against *E. amylovora* showed that *P. syringae* had no inhibitory activity against *E. amylovora* under the same media conditions tested (data not shown). These results suggest that if the cluster in ES4326 also produces an antimicrobial compound, it may be differentially regulated, such that it is not induced under the same conditions as PNP-1. Alternatively, the chemical structure and/or spectrum may be different than PNP-1, due to the differing composition of their gene clusters.

The chemical nature of the PNP-1 antibiotic is still not known. The homologous cluster in *P. chlororaphis* PCL1391 directs the production of a phenazine antibiotic, phenazine-1-carboxamide (Figure 1). Phenazines are heterocyclic compounds with various residue substitutions around the rings, and as a result produce a range of pigments from dark red to bright yellow to bright blue [54]. Those that are antimicrobial tend to be broad-spectrum, and have many ecological roles [55]. Phenazine-1-carboxamide (PCN) from *P. chlororaphis* PCL1391 has been shown to inhibit the effects of tomato foot and root rot, caused by *Fusarium oxysporum* [56]. In addition, PCN enhances *P. chlororaphis* colonization of the rhizosphere by eliminating all competition for nutrients and space [57]. Although this cluster is homologous to that of PNP-1, PNP-1 does not appear to be broad-spectrum like PCN. *P. agglomerans* EH1087 has been reported to produce a phenazine antibiotic; D-alanylgriseoluteic acid (AGA) [24], [58]. AGA, however, is a broad-range antibiotic effective against a wide range of Gram-negative and Gram-positive bacteria, unlike PNP-1 [24]. In addition, AGA synthesis has been attributed to a sixteen gene cluster, approximately 14 kb in size [24], and is not homologous to the PNP-1 biosynthetic cluster. Characteristic of a phenazine antibiotic, the activity of both AGA and PNP-1 is not abolished in the presence of amino acids [24].

This work has identified an antibiotic in *P. ananatis* BRT175 whose biosynthesis is determined by an 8.2 kb biosynthetic gene cluster consisting of seven genes. The loss of antibiotic production and/or export in 24 independent transposon mutants is strong evidence that the seven-gene cluster identified is likely responsible for the production of the antibiotic. Although this cluster has yet to be reported within *Pantoea*, the identification of homologous antibiotic biosynthetic gene clusters in *Pseudomonas* that have been shown to produce an antimicrobial phenazine adds further support to our conclusion. Although there are several conserved genes between this cluster and those of the pseudomonads, differences in the genetic composition of the clusters, along with the spectrum of activity, suggest that PNP-1 is likely structurally and functionally different from phenazines. Still, further characterization of the gene cluster, including its role in antibiotic biosynthesis, and the determination of the biosynthetic steps and chemical structure of PNP-1 are presently being pursued. PNP-1 has the potential to be used to control *E. amylovora*, as well as clinically and agriculturally relevant *Pantoea* isolates.

## Supporting Information

Figure S1 **Effects of nutrition on antibiotic production.** A) LB medium with and without glucose, B) *E. coli* minimal medium, with and without glucose; C) *E. coli* minimal medium with varying peptone concentrations, with and without glucose; D) *E. coli* minimal medium with varying tryptone concentrations, with and without glucose.
(DOC)
