# Supplementary material for: Identification of a Pantoea Biosynthetic Cluster That Directs the Synthesis of an Antimicrobial Natural Product
Source: PLoS One. 2014 May 5;9(5):e96208. doi: 10.1371/journal.pone.0096208 (PMC4010436; doi:10.1371/journal.pone.0096208)
Supplement: Figure S1 — Effects of nutrition on antibiotic production. A) LB medium with and without glucose, B) E. coli minimal medium, with and without glucose; C) E. coli minimal medium with varying peptone concentrations, with and without glucose; D) E. coli minimal medium with varying tryptone concentrations, with and without glucose. (DOC) [file pone.0096208.s001.doc]

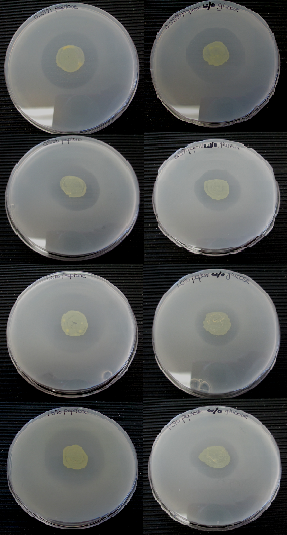

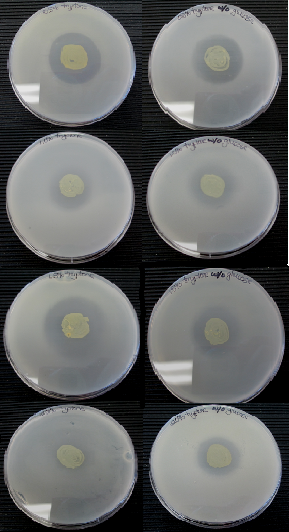

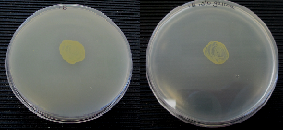

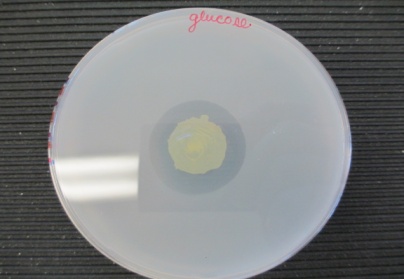

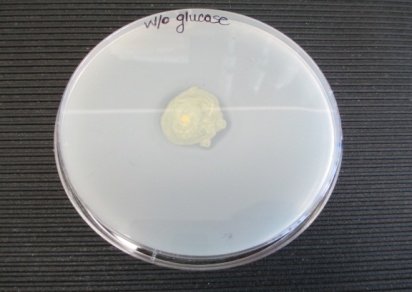


with glucose

with glucose

with glucose

without glucose

without glucose

without glucose

with glucose

without glucose

A)

B)

C)

**0.25%**

**0.5%**

**1.0%**

**1.5%**

**0.5%**

**1.0%**

**1.5%**

**2.0%**

D)

Figure S1. Effects of nutrition on antibiotic production. A) LB medium with and without glucose, B) *E. coli* minimal medium, with and without glucose; C) *E. coli* minimal medium with varying peptone concentrations, with and without glucose; D) *E. coli* minimal medium with varying tryptone concentrations, with and without glucose.
